# Combining Random Forests and a Signal Detection Method Leads to the Robust Detection of Genotype-Phenotype Associations

**DOI:** 10.3390/genes11080892

**Published:** 2020-08-05

**Authors:** Faisal Ramzan, Mehmet Gültas, Hendrik Bertram, David Cavero, Armin Otto Schmitt

**Affiliations:** 1 Breeding Informatics Group, Department of Animal Sciences, Georg-August University, Margarethe von Wrangell-Weg 7, 37075 Göttingen, Germany; faisal.ramzan@stud.uni-goettingen.de (F.R.); gueltas@informatik.uni-goettingen.de (M.G.); hendrik.bertram@stud.uni-goettingen.de (H.B.); 2 Department of Animal Breeding and Genetics, University of Agriculture Faisalabad, 38000 Faisalabad, Pakistan; 3 Center for Integrated Breeding Research (CiBreed), Albrecht-Thaer-Weg 3, Georg-August University, 37075 Göttingen, Germany; 4 H&N International, 27472 Cuxhaven, Germany; cavero@ltz.de

**Keywords:** Random Forests, signal detection, genome wide association studies, boruta, eggshell strength, egg weight

## Abstract

Genome wide association studies (GWAS) are a well established methodology to identify genomic variants and genes that are responsible for traits of interest in all branches of the life sciences. Despite the long time this methodology has had to mature the reliable detection of genotype–phenotype associations is still a challenge for many quantitative traits mainly because of the large number of genomic loci with weak individual effects on the trait under investigation. Thus, it can be hypothesized that many genomic variants that have a small, however real, effect remain unnoticed in many GWAS approaches. Here, we propose a two-step procedure to address this problem. In a first step, cubic splines are fitted to the test statistic values and genomic regions with spline-peaks that are higher than expected by chance are considered as quantitative trait loci (QTL). Then the SNPs in these QTLs are prioritized with respect to the strength of their association with the phenotype using a Random Forests approach. As a case study, we apply our procedure to real data sets and find trustworthy numbers of, partially novel, genomic variants and genes involved in various egg quality traits.

## 1. Introduction

The importance of genotype-phenotype association studies to understand the genetic basis of traits, either qualitative or quantitative, is well established [1]. Single-SNP based models that test individual SNPs for their association with the phenotype in a genome wide association study (GWAS) are widely used in this regard. Although this approach has been quite successful in discovering genes affecting important traits [2], some daunting aspects still persist that reduce its power at best and make it error prone at worst. These inherent features include population stratification or relatedness among the samples, multiple hypothesis testing, and overestimation of SNP effects as pointed out in previous studies [3,4,5,6]. Linear mixed model (LMM) based approaches that incorporate the covariance structure across individuals have been found most effective in dealing with both the kinship and the population stratification problem [7,8,9,10]. Acknowledging their importance, a series of approaches have been proposed to implement the LMM in the context of GWAS [11]. Similarly, many multiple testing correction methods with varying strictness have been suggested as possible solutions and some of these have been addressed in References [4,12].

A further challenge in analyzing quantitative traits is to discover loci having moderate to small phenotypic effects. The SNPs present either inside or in the vicinity of these quantitative trait loci (QTLs) display association strengths which are too small to exceed the statistical significance threshold value. Consequently, only a small part of the overall variance is captured in a typical GWAS analysis [13]. Haplotypes can capture the correlation structure of SNPs which is ignored in single-SNP based GWAS approaches. Hence, testing the haplotypes for association looks promising at least in theory. Nevertheless, haplotype based analyses are far from being simple and so far, no clear evidence is available in the literature that the haplotype based tests are more powerful than single-SNP based tests even though this topic has been investigated over the years [14,15,16,17]. To address these limitations, multi-SNP GWAS models were introduced that fit all SNPs simultaneously as random effects in the model [18]. Many implementations of multi-SNP models based on Bayesian as well as LMM frameworks have been developed [19]. Numerous studies have also been conducted to show comparative performance of different single-SNPs, haplotype and multiple-SNP models along with their different implementations [11,20,21,22,23,24]. Recently, the growing application of machine learning approaches in different fields of science has incited their use in assessing the genotype-phenotype association as well [25,26,27,28,29]. Multiple studies have confirmed the superiority of machine learning algorithms compared to GWAS approaches by identifying genes having small effects on the phenotype [26,29,30]. Machine learning methods do not require prior assumptions about the distribution of the SNP effects, hence can be used for a wide variety of traits in humans [31], plants [28] and livestock [32,33]. In particular, Random Forests (RF) models have been praised for their ability to analyze a large number of loci simultaneously and to identify promising associations [29,30].

All the above mentioned methodologies have their advantages and challenges. Among other factors, the success of different association methods is heavily influenced by the genetic architectures of the trait of interest [24,34]. Given the complexity underlying the genetics of quantitative traits, it is probably not realistic to assume that any one method can retain its statistical power for different genetic architectures [17,35,36]. Single-SNP based models are still popular [37,38,39,40,41] while the RF based methods are gaining importance [42]. However, an increasing number of scientists are recommending the integration of different association methods in order to improve QTL identification and interpretation [43,44]. In this regard, to bridge the gap between single-SNP and haplotype based analysis, Zhang et al. [45] used a non-parametric spline based technique to integrate multiple single-SNP based test statistics into a single test. Furthermore, Zhang et al. [19] as well as Abed and Belzile [24] suggested the combined usage of single-SNP and multi-SNP methods together for the identification of a robust set of SNPs associated with the complex phenotypes. To combine the advantages of machine learning and parametric GWAS analysis, Nguyen et al. [26], Huang et al. [28] and Schwarz et al. [46] employed a two stage analysis integrating the Random Forests algorithm with single-SNP models. However, the selection of SNPs in one stage and the analysis of the selected SNP in the second step may not account for the hidden structure in the data and can result in inflated SNP effects in the discovery of genotype-phenotype association.

In this study, we propose a framework that mainly focuses on the identification of robust genotype-phenotype association signals by combining the important SNPs obtained in different association analyses. For this purpose, we first perform a signal detection strategy using the test statistic values of single-SNP based GWAS analysis for the detection of QTLs. Second, using a Random Forests based feature selection technique, we assess the relative importance of SNPs regarding their association level with the phenotype. Unlike the previous two stage studies [26,28,46], we finally prioritize the important SNPs within the QTLs to discover the most robust set of markers.

In order to demonstrate the functionality of our framework, we have analysed two different GWAS (genotype-phenoype) datasets in this study. The first dataset contains the eggshell strength (ESS) measured at two different time points during the productive life of chicken and the second dataset is related to egg weight (EW) in chicken. Our results show that, using our framework, we are able to identify important novel markers/genes which could provide new insights into the genetic architecture of these traits.

## 2. Materials and Methods

### 2.1. Data Sets

In this study, we have analysed two chicken datasets to detect genotype-phenotype associations underlying economically important egg quality traits, namely eggshell strength (ESS) and egg weight (EW).

**Dataset 1:** The first dataset contains eggshell strength recorded at two time points during the lifetime of the birds. We have previously used this dataset in Reference [30] to identify the key regulatory mechanisms governing eggshell strength in chicken. The dataset consists of 892 birds from six generations of a purebred commercial brown layer line genotyped with the Affymetrix Axiom^®^ 600 K Chicken Genotyping Array. The corresponding phenotypic data contain de-regressed breeding values of eggshell breaking strength from individual birds at two different stages of production. The eggshell strength was measured at the poles of an egg and represents the force in Newton needed to break the egg. For the first time point, ESS was recorded at the age of 42, 45, and 48 weeks, while for the second time point, recordings were made at the age of 64 and 68 weeks. Average values of the recorded breaking strengths at time point 1 (ESS1) and time point 2 (ESS2) were then used in an animal model for the breeding value estimation. In this analysis, we also used pedigree data consisting of 40,545 individuals from six generations, in total. The estimated breeding values were then de-regressed following Garrick et al. [47] to obtain the pseudo-phenotypes that were then used for the further analysis. To ensure the quality of our data, we filtered the genotypic data to remove the SNPs having minor allele frequency ≤0.01, genotyping call rate ≤97% and also those deviating from Hardy–Weinberg equilibrium (*p*-value < 1 × 10${}^{-6}$). Birds having a SNP call rate smaller than 95% were also removed. Finally, we had 892 animals and 318,513 SNPs for our analyses.

**Dataset 2:** The second dataset pertains to egg weight recorded in 36 weeks old adult birds. The dataset has been previously analysed to perform GWAS of age dependent egg weights (EW) in chicken [48]. The dataset provides genotypes and phenotypes of 1063 birds belonging to a pure bred line of Rhode Island Red chicken, also genotyped with the Affymetrix Axiom^®^ 600 K Chicken Genotyping Array. From the seven age levels analysed in the original study, we re-analysed only EW at 36 weeks of age as the most significant associations were reported for this trait. The genotypic data were filtered for SNP call rates, minor allele frequencies and Hardy Weinberg equilibrium using the same threshold values as given for the first dataset. After filtering, we used 294,705 SNPs and 1036 birds in our analysis.

### 2.2. Analysis Framework

Our proposed analysis framework consists of six phases to detect important SNPs associated with phenotypes under study.

**Phase 1:** Following the study of Liu et al. [48], we perform a GWAS to obtain the association between single-SNPs and the phenotypes. For this analysis, we first applied a principal component analysis (PCA) using the independent SNPs obtained after pruning SNPs using the indep-pair-wise option in PLINK [49] software, with a window size of 25 SNPs, a step of 5 SNPs and a $r^{2}$ threshold of 0.2. Then we used the top five of those principal components as covariates in the association model to control for population structure. Next, we performed a GWAS analysis based on the following univariate linear mixed model implemented in the FaST-Lmm v0.2.31 software [50].

(1) y=Wα+xβ+u+ϵ.

**Figure 1:**
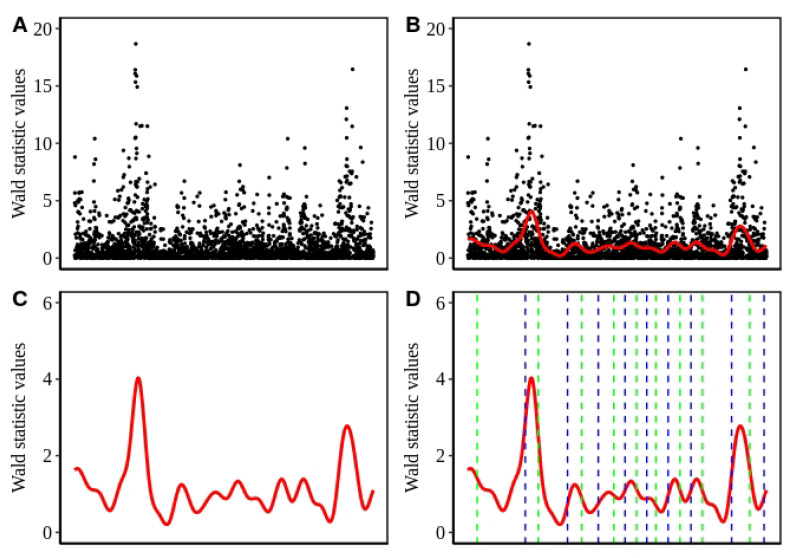
Step by step representation of the peak detection method. (**A**) Distribution of the test statistic values along the length of a chromosome segment. (**B**) The red line indicates the cubic spline fitted on the test statistic values represented by the black dots. (**C**) The same cubic spline curve as in B without points, y-axis rescaled (**D**) Dashed lines represent the inflection points of the curve. A pair of a left (blue) and a right (right) inflection point constitute a peak.

In Equation (1), y is the vector of phenotypic values for all individuals; W is the matrix of covariates; α is a vector of corresponding effects and the intercept; x is the vector of genotypes for the SNPs tested; β is the effect size of the marker; u is a vector of random polygenic effects with a covariance structure as $u∼N(0,KV_{g})$, where K represents the genetic relatedness matrix derived from the SNP markers and $V_{g}$ is the polygenic additive variance. ϵ is the vector of random residuals with $\epsilon ∼N(0,IV_{e})$, where I is the identity matrix and $V_{e}$ is the residual variance component. To test the value of β for each SNP against the null hypothesis $H_{o}:\beta =0$, the Wald-test $\left(F_{\mathrm{Wald}}=\hat{\beta^{2}}/\mathrm{Var}\left(\beta \right)\right)$ was applied. As suggested in Reference [48], the adjusted threshold value was determined using the *simpleM* approach [51] to evaluate the significance of individual SNPs. In Figure 1A we exemplarily show a chromosomal region and its corresponding Wald statistic values.

**Phase 2:** For the elaboration of association signals embedded in the Wald test statistics, we apply a cubic smoothing spline on these values. The cubic smoothing spline is a piece-wise defined cubic function and is based on the same principle as the normal cubic regression. The assumption implicit in this approach is that the individual association values are observed with noise and that these values can be considered as estimations of some underlying function *g*. Given the marker positions in the genome $\left(x_{i}\right)$ and the corresponding association values $\left(y_{i}\right)$, the function *g* is estimated by minimizing the following expression

(2) $$S\left(f\right)=\sum {\left\{y_{i}-g\left(x_{i}\right)\right\}}^{2}+\lambda \int g^{\prime \prime }{\left(x\right)}^{2}dx.$$

In Equation (2), the first part of right hand side represents the residual sum of squares with the cubic spline function $g(x_{i})$ being the estimated value of the function *g* corresponding to SNP *i* at chromosomal position $x_{i}$. The integral represents a roughness penalty controlled by the tunable parameter λ whose value is determined by cross validation. The penalty controls the trade off between the conflicting goals of matching the given data and producing a smooth curve [52]. $g^{\prime \prime }$ represents the second derivative of the sought function with respect to *x*. The assumption of *g* being continuous and twice differentiable leads to its approximability via a cubic smoothing spline [53]. Thus, a continuous and smooth curve, suitable for the elaboration of the association signals in the test statistic values is obtained. In Figure 1B,C, we exemplarily show the application of cubic smoothing spline over the Wald statistics in a small chromosomal region.

**Phase 3:** For delineation of the obtained association signals in the form of peaks, we determined the inflection points based on the smoothed values. As the smoothing curve represents a function g(x), the inflection points indicate the positions, where $g^{\prime \prime }\left(x\right)=0$ and thus the curve changes its curvature. Hence, the region between two consecutive inflection points having a downward concave form is regarded as a peak. To this end, the maximum value within a peak is recorded as the height of the peak. In Figure 1D, we exemplarily show the identified peak regions based on the inflection points.

**Phase 4:** In order to separate the peak regions having association signals higher than those arose by chance, we have created a null distribution by permutating the phenotypic data. For the construction of the null distribution, Phases 1, 2 and 3 have been applied to each permutated dataset and the maximum peak values were recorded. In our analysis, we permutated the dataset 1000 times. In the real dataset, we defined a peak region as a QTL if the corresponding peak height exceeds the 95th percentile of the null distribution.

**Phase 5:** Adopting the strategy from our previous study [30], the Random Forests (RF) algorithm was used to estimate the relative importance of each SNP (attribute) for the prediction of the response variable (phenotype). For this purpose, we applied the Boruta algorithm [54] which is a powerful wrapper for the RF based feature selection approach to assess the importance of SNPs. Consequently, we obtained a decision for each SNP whether the importance of the SNP is confirmed, rejected or tentative. In our analysis we only considered SNPs with confirmed importance.

**Phase 6:** Finally, to prioritize the SNPs which are in the QTLs detected in Phase 4, we use the important SNPs from Phase 5 and define the SNPs discovered in both Phases as robust SNPs in our analysis.

### 2.3. Extraction of the Candidate Genes

We scan the genome to identify the genes corresponding to the robust SNPs using BioMart [55]. Only those genes were considered to have some association with the phenotype that were harboring at least one of the robust SNPs within its boundaries. The R-script used for this analysis is provided in Supplementary File S1.

## 3. Results

In our study, we suggest an analysis framework to improve the power of commonly implemented GWAS. The overall framework comprises the following steps. First, a linear mixed model (LMM) based single-SNP GWAS is performed to obtain test statistics representing the strength of association between each SNP and the phenotype. Second, performing the signal detection strategy by fitting a cubic smoothing spline on the test statistic values, we identify QTLs. Third, we apply the RF classifier using the Boruta algorithm to assess the relative importance of SNPs regarding the level of their association with the phenotype. Finally, the important SNPs are prioritized within those QTLs to discover a robust set of SNPs associated with the phenotype. Two different GWAS (genotype and phenoype) datasets related to eggshell strength (ESS) and egg weight (EW) have been analysed using this framework to demonstrate its functionality.

### 3.1. Single-SNP Based GWAS Analysis

**Figure 2:**
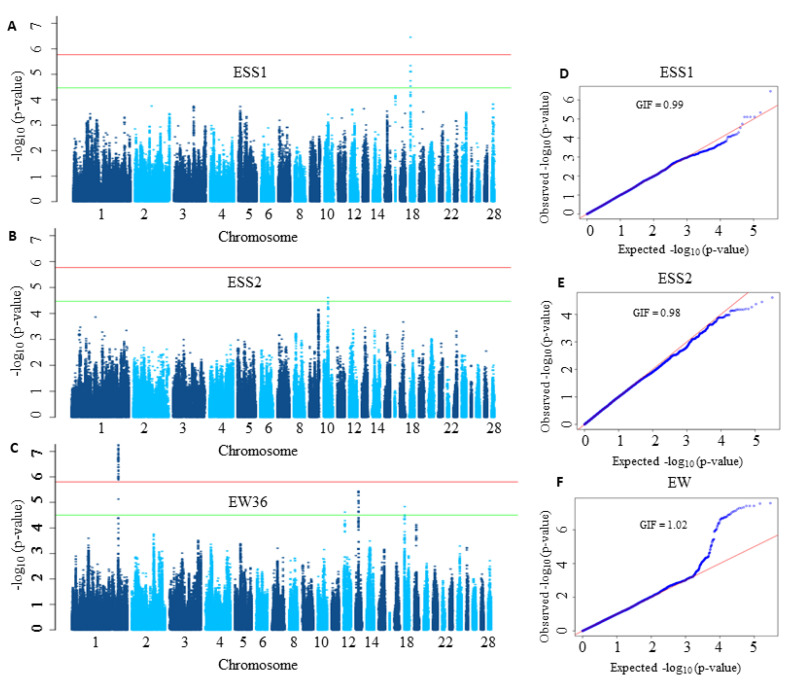
Manhattan and Q-Q plots corresponding to eggshell strength at time point 1 (ESS1), time point 2 (ESS2) and egg weight at 36 weeks of age (EW36). In Manhattan plots (**A**–**C**), the horizontal red and green lines denote the genome-wide significance (*p*-value = $1.7\times 10^{-6}$ for ESS1 and ESS2 and $1.5\times 10^{-6}$ for EW36) and suggestive significance thresholds (*p*-value = $3.4\times 10^{-5}$ for ESS1 and ESS2 $3.1\times 10^{-5}$ for EW), respectively. The −log10 of the observed *p*-values for each single nucleotide polymorphism (SNP) is given on the y-axis while its position on a chromosome is given on the x-axis. In Q-Q plots (**D**–**F**) the observed −log10 transformed *p*-values are plotted against the expected −log10 transformed *p*-values. GIF stands for genomic inflation factor.

In order to demonstrate the limited power of conventional single-SNP based GWAS analysis, we first analysed both datasets using a LMM as suggested for the analysis of egg weight (EW) in the study of Liu et al. [48]. In the application of LMM, we considered the correction of the population stratification and applied the *SimpleM* method [51] for multiple testing correction. The LMM approach for eggshell strength (ESS) at time point 1 (ESS1) and time point 2 (ESS2) led to the identification of only one significant SNP for ESS1 (see Figure 2A,B). Furthermore, the LMM method revealed 43 significant SNPs for EW (see Figure 2C) on chromosome 1 (GGA1) which were then mapped to three genes (ITM2B, RCBTB2, RB1).

Today it is well known that quantitative traits are influenced by a large number of genes mostly having small effects. But as shown in Figure 2, many association signals were not strong enough to reach the significance threshold, thereby their influences on the phenotype are missed.

### 3.2. Detection of Genotype-Phenotype Association Using the Combined Framework

To identify genes showing weak association signals that remain undetected in the typical GWAS analysis, we applied our analysis framework to both datasets.

The analysis of the ESS datasets reveals eight QTLs for ESS1 and five QTLs for ESS2 based on the signal detection approach. The details of these QTLs are given in Table 1. Interestingly, we found chromosome 9 (GGA9), 10 (GGA10), 15 (GGA15) and 20 (GGA20) to have QTLs associated with ESS at both time points. Especially, the QTLs on GGA20 are overlapping and underpin the same genomic region as associated with ESS at both time points. In addition, the application of the RF classifier provides 3726 and 1815 SNPs which map to 405 and 253 genes associated with ESS1 and ESS2, respectively. The lists of these SNPs and their corresponding genes are taken from our previous study [30]. The investigation of these SNPs in the identified QTLs reveals 158 and 14 robust SNPs related to ESS1 and ESS2, respectively (the list of the SNPs is given in Table S1).

**Figure 3:**
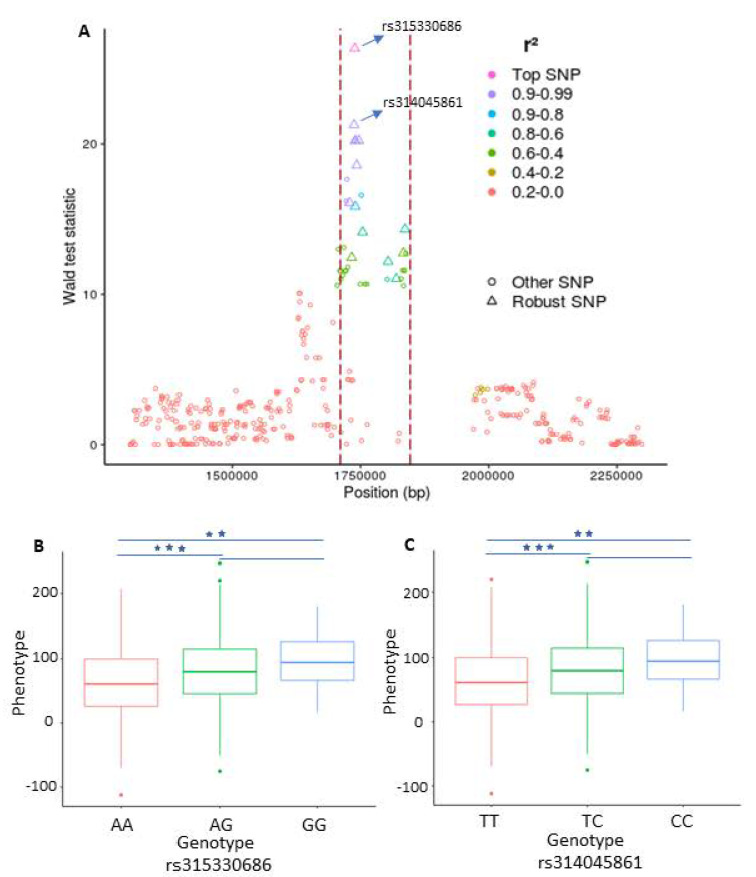
Plot representing a genomic region on chromosome 18 which is in association with eggshell strength at time point 1 (ESS1). (**A**) Plot representing the linkage disequilibrium (LD) structure inside and around a significant peak. The dotted red lines depict the boundaries of the peak. Each point represents a single nucleotide polymorphism (SNP) and the color shows the strength of LD between the top SNP inside the peak and the SNP surrounding it. The diamond shape points inside the peak depict the robust SNPs. The X-axis contains the SNP positions on the chromosome while the y-axis depicts the Wald statistic values obtained from the single-SNP based genome wide association study (GWAS) analysis. (**B**,**C**) The effects of different genotypes of the two leading SNPs identified in the combined framework for ESS and their significance (${}^{**}\;p<0.01$, ${}^{***}\;p<0.001)$.

Of particular interest here is the LD analysis that we performed based on the robust SNPs to further elaborate their makeup in the identified QTLs. The LD analysis reveals, as expected, that the robust SNPs inside the QTLs have a remarkably higher level of LD than the surrounding SNPs (see Figure 3A). To this end, we exemplarily compared the phenotype differences between the genotypes of the top two SNP (rs315330686, rs314045861) on GGA18. The comparison suggests that for both SNPs, the birds homozygous for the minor alleles have higher phenotypes than those of the other two genotypes (Figure 3B,C).

The extraction of the genes corresponding to the robust SNPs reveals 14 and 3 genes for ESS1 and ESS2, respectively (the list of the genes is given in Table S1). The functional investigation of these genes shows that the majority of them were annotated to play essential roles in the transport of minerals and organic compounds. Seven of these genes, namely ATP6V0A2 (ATPase, H+ Transporting, Lysosomal V0 Subunit A2), DDX55 (DEAD-Box Helicase 55), DNAH10 (Dynein Axonemal Heavy Chain 10), GTF2H3 (General Transcription Factor IIH Subunit 3), MYO1E (Unconventional Myosin 1E), TCTN2 (Tectonic Family Member), and MYH10 (Myosin Heavy Chain 10)), have molecular functions related to the activity of the ATPase enzyme. Interestingly, in relation to eggshell formation ATPases have long been known to show intense activity in the cells of shell gland during the synthesis of eggshell [56]. Furthermore, CHRNA7 (Cholinergic Receptor Nicotinic Alpha 7 Subunit), is associated with the transport of ions, especially calcium ions. The other main function performed by the identified genes includes cell morphogenesis which ensures the homeostasis of tissues involved in the development of eggshell [57,58]. The genes that play a role in this process include NDEL1 (NudE Neurodevelopment Protein 1 Like 1), ADGRB1 (Adhesion G Protein-Coupled Receptor B1), THSD4 (Thrombospondin Type 1 Domain Containing 4) and EIF2B1 (Eukaryotic Translation Initiation Factor 2B Subunit Alpha).

Among the genes found to be associated with ESS2, TRPM7 (Transient Receptor Potential Cation Channel Subfamily M Member 7) and BNC1 (Basonuclin 1) have functions related to the homeostasis of ions in the cell. On the other hand, the CDH4 (Cadherin-4) gene that was found for both ESS1 and ESS2 encodes for R-cadherin/cadherin-4 which are single-chain integral membrane glycoproteins and mediate calcium-dependent cell—cell adhesion. Reduced levels of these cell adhesion molecules lead to the age-related decline in tissue homeostasis [59]. Along with other members of the cadherin superfamily, R-cadherins play roles in cell differentiation in a variety of tissues including bones, kidneys and uterus [60,61,62,63].

The analysis of the EW dataset resulted in the detection of eleven QTLs including the one revealed on chromosome 1 (GGA1) in the original study [48]. The additional QTLs were found on chromosomes 4 (GGA4), 12 (GGA12), 13 (GGA13), 14 (GGA14), 15 (GGA15) and 18 (GGA18). The details of these eleven QTLs are summarized in the Table 2. Remarkably, there is no overlap between the QTLs observed for EW and ESS. The application of the RF classifier on this dataset provides a list of 753 important SNPs. A closer look at these SNPs points out that 145 of them (including 41 SNP identified in the original study [48]) are defined to be robust SNPs due to their genomic positions within the QTLs (the list of the SNPs is given in Table S1). Similar to the analysis of the ESS dataset, LD analysis based on the EW dataset also demonstrates the presence of strong linkage between robust SNPs.

The extraction of the genes associated with the robust SNPs related to EW results in the determination of 16 genes (the list is given in Table S1). Despite no overlap between the QTLs identified for ESS and EW, a variety of genes are involved in the same biological functions. Especially, many of the genes have their functions annotated to trans-membrane transportation of minerals and proteins. In this regard, genes including SCNN1G (Sodium Channel Epithelial 1 Subunit Gamma), AFAP1L1 (Actin Filament Associated Protein 1 Like 1), CD99L2 (CD99 Molecule Like 2), GPR50 (G Protein-Coupled Receptor 50), GRIA2 (Glutamate Ionotropic Receptor AMPA Type Subunit), GRPEL2 (GGrpE Like 2, Mitochondrial), HS3ST4 (SH3 Domain And Tetratricopeptide Repeats 2), ITM2B (Integral Membrane Protein 2B), MED4 (Mediator Complex Subunit 4), MTMR1 (Myotubularin Related Protein 1) and SH3TC2 (SH3 Domain And Tetratricopeptide Repeats 2) encode proteins that are part of cell membranes. By regulating the transport of ingredients for the egg development they can play a role in the determination of EW. More importantly, the SCNN1G encodes a non-voltage gated sodium channel to ensure the trans-membrane transportation of sodium ions. Higher expression of this gene during egg formation has been reported to play an important role in the determination of eggshell quality [64]. Similarly, the GRIA2 gene product functions as ligand-activated cation channel that allows the trans-membrane transportation of different ions. On the other hand, genes like RCBTB2 (RCC1 and BTB Domain Containing Protein 2) and TBC1D8B (TBC1 Domain Family Member 8B) can play a role in the regulation of these transportation channels. Functional annotations of RB1 (Retinoblastoma Transcriptional Corepressor 1) and MED4 genes are related to nuclear hormone receptor binding, a process principally involved in mineral metabolism. In particular, the MED4 encoded protein is a component of the vitamin D receptor-interacting protein complex that has been shown to contribute critically for the regulation of calcium absorption in the intestine [65]. The regulation of the intra-cellular protein transport and the cellular protein localization are biological functions performed by the ABLIM3 (Actin Binding LIM Protein Family Member 3) gene.

## 4. Discussion

Deciphering genotype-phenotype associations for quantitative traits still remains challenging due to the weak contribution of many individual SNPs to the phenotype. To address this problem, several approaches including single-SNP or multiple-SNP based models have been developed [18]. The worth of single-SNP models is well testified by the repertoire of genes related to a variety of traits that has been discovered using these models [2]. However, for quantitative traits where a multitude of genes may act in concert to confer a particular phenotypic value to an individual, the power of these single-SNP based models is limited [4,6,30,66]. Multi-SNP models are potentially more competent for the detection of smaller effects, but mostly require a prior distribution of SNP effects that is not known for most of the traits while for some traits they may not even follow a strict distribution [18,67]. To overcome these limitations, combining single-SNP based statistics over a genomic region to test its association with the trait has been the method of choice for many scientists [68,69,70,71]. In this regard, Beissinger et al. [72] show the superiority of cubic smoothing spline techniques over some other methods to combine single-SNP based statistics for the discovery of selection signatures. Furthermore, Zhang et al. [45] have praised the utility of spline based techniques to integrate association statistics in order to identify the causal alleles. However, these methods do not provide a clear framework that can be used to identify genomic regions with subtle effects on the phenotypes in samples with family or population structures.

With the growing application of machine learning algorithms in the field of genomics, their application to ascertain the genotype-phenotype association is gaining importance. Contrary to traditional multi-SNP models, machine learning methods do not require any prior assumptions about the genetic architecture of traits. In our recent study [30], we successfully applied an RF classifier to the ESS dataset to assess the importance of SNPs and identified large numbers of genes associated with eggshell strength at two different production stages. Despite the success of the RF classifiers in association analysis, there is still a need to prioritize the identified genes to recognize the genes having most robust association with the phenotype. This prioritization constitutes a means to delve deeper into the functioning of the individual genes to understand their marginal influences on the manifestation of the phenotype differences among the samples. For this purpose, we investigated genes within the QTLs that have association signals higher than expected by chance. The identification of QTLs is a fundamental step in our study which we have performed using a splines based strategy in several phases. Unlike previous studies [45,72], using this technique we harness the association signals, in order to detect the genomic regions harbouring genes potentially playing roles in the phenotype manifestation.

**Figure 4:**
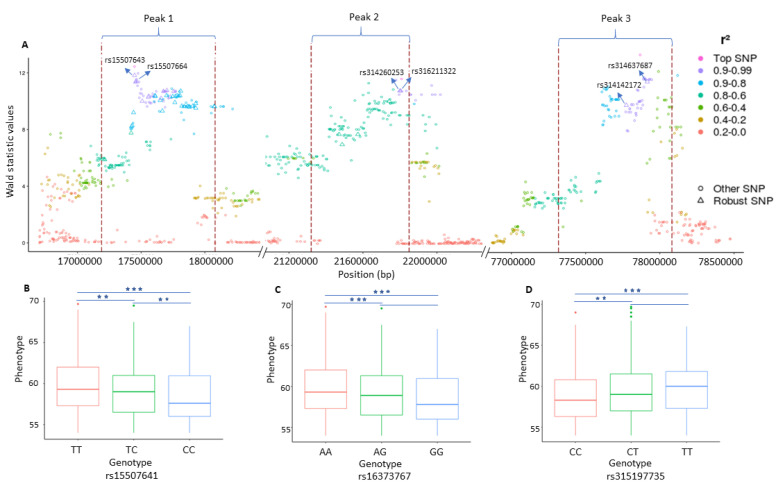
Plot representing three genomic regions on chromosome 4 in association with egg weight (EW). (**A**) Plot representing the LD structure inside and around the significant peaks. The dotted red lines depict the boundaries of the peaks. Each point represents a SNP and the color shows the strength of linkage disequilibrium (LD) between the top single nucleotide polymorphisms (SNPs) inside each peak and the surrounding SNPs. The diamond shape points inside the peak depict the robust SNPs. The X-axis contains the SNP positions on the chromosome while the y-axis depicts the Wald statistic values obtained from single-SNP based GWAS analysis. ((**B**–**D**) The effects of different genotypes of the three leading SNPs identified for EW and their significance (${}^{**}\;p<0.01$, ${}^{***}\;p<0.001)$).

Our results show that the determination of QTLs by our signal detection approach and then the prioritization of SNPs within these QTLs (called robust SNPs), can lead to the discovery of genes which despite having association to the phenotypes, remain undetected in the typical GWAS. Especially, the combined usage of both methods (RF and signal detection) not only identify the QTLs having small effects but also helps to identify the SNPs in those QTLs that had their association value higher than expected by chance. (see Figure 3A and Figure 4A). Moreover, the LD based on the robust SNPs (Figure 3A and Figure 4A) supports us, on the one hand, to monitor their strong mutual correlation which is crucial to explain the genetic makeup of the underlying QTLs. On the other hand, it further substantiates our idea regarding the presence of signals which are caused by the strong LD in the QTLs and embedded in the association statistics.

Although both of the traits analysed in this study were related to egg quality, the identified genes are distinct for ESS and EW in this study. This distinction was expected as the chickens genotyped in the two datasets have different genetic backgrounds. Remarkably, however some of these genes are involved in the same biological function related to transmembrane transportation of elements including minerals and organic compounds. Further, the majority of the ESS1 related genes are responsible for the availability of calcium (Ca^2+^) and bicarbonate (HCO^3−^) which are prerequisites for eggshell mineralization in the uterus part of the oviduct. These ions are supplied in large amounts via trans-epithelial transport in the uterus, for which ion channels, ion pumps and ion exchangers are required [73]. This function is mainly regulated by ATPase, an enzyme which is implicated in this process through several genes which were identified in this analysis for ESS. The ATPase enzyme decomposes ATP into ADP to release the energy required to perform energy intensive tasks by the cell. Regarding eggshell formation, ATPases have long been known to influence the microvilli of the tubular cells of the shell gland during the process of eggshell formation [56]. Similarly, inhibition of ATPase from the shell glands has been demonstrated to cause the thinning of the eggshell due to the inhibition of the calcium transport across the shell gland epithelium which is known to be an energy expensive process [74]. The hydrogen potassium ATPase maintains a certain pH level of the uterine fluid during the eggshell formation by acting as a pump to transfer the hydrogen ions (H^+^) from the uterine cell of chicken to plasma. In this regard, two paralogs (ATP6V1B, ATP6V1C2) of the ATP6V0A2 gene found in our study have been previously reported to transfer hydrogen ion from chicken uterine cells to blood plasma during the process of egg calcification [73,75]. When integrated into biological membranes, the so-called transmembrane ATPases take part in the transportation of metabolites across the membranes [76]. Transmembrane ATPases exchange many metabolites across the membranes and provide the necessary environment for activities of the cell [77]. Similarly, genes discovered for EW encode cell membrane proteins which can act as channels for the transportation of minerals as well as proteins. Among these, one of the most important channel protein is encoded by the SCNN1G. This gene belongs to the sodium channel gene family. Many members of this gene family are known to affect egg weight as well as other egg quality traits [64].

The other important functional category that many of the genes related to ESS could be linked to is cell morphogenesis. Previous studies presenting the transcriptome profile of different segments of the chicken oviduct have also reported a large number of genes annotated for functions related to morphogenesis [73,78,79]. It is also important to note the difference in genes identified for ESS1 and ESS2. It depicts the change in the genetic and environmental components of the phenotypic variance over age which has been previously reported for other complex traits [80,81]. Given all these results, our suggested framework is capable of highlighting the important genes within the QTLs having moderate to small effects. The availability of larger datasets can further improve the power of this framework to detect novel QTLs. Furthermore, well established polygenic approaches can also be integrated in this framework for the discovery of even robust associations. On top of that, our strategy is complementary to our previous study in which we performed a RF based feature selection technique for genotype-phenotype association.

## Figures and Tables

**Table 1:** Significant peaks as defined in Phase 4 of our analysis framework and corresponding quantitative trait loci (QTLs) for ESS1 and ESS2.

Chromosome	No. of SNPs	Start Position	End Position	No. of Genes	Trait
2	204	147,575,318	148,273,465	3	ESS1
9	66	21,762,694	21,953,310	0	ESS1
9	82	21,777,888	22,001,729	0	ESS2
10	75	6,517,673	6,728,897	4	ESS1
10	86	9,922,422	10,054,824	2	ESS1
10	60	10,715,120	10,818,097	3	ESS2
10	61	11,245,585	11,351,799	1	ESS2
12	112	10,948,518	11,227,521	2	ESS1
15	42	4,908,007	5,006,688	7	ESS1
15	43	6,193,090	6,273,778	3	ESS2
18	38	1,722,586	1,836,741	2	ESS1
20	51	7,589,607	7,717,177	1	ESS1
20	46	7,599,368	7,711,505	1	ESS2

**Table 2:** Significant peaks as defined in Phase 4 of our analysis framework and corresponding QTLs for EW.

Chromosome	No. of SNPs	Start Position	End Position	No. of Genes
1	304	167,931,038	169,505,140	25
4	205	17,189,770	18,080,445	9
4	143	21,319,808	21,849,558	3
4	136	77,317,446	78,081,369	4
12	39	2,849,562	3,010,032	7
13	49	8,495,533	8,608,578	6
14	58	7,023,793	7,188,250	4
15	41	11,193,342	11,309,808	8
15	35	11,419,957	11,514,516	3
18	30	1,057,714	1,136,220	1
18	28	1,179,899	1,238,583	0

## Supplementary Materials

The following are available online at https://www.mdpi.com/2073-4425/11/8/892/s1, Script S1: R-script for analysis of SNPs and for the extraction of corresponding genes, Table S1: The list of important SNPs and genes, Figure S1: Venn diagrams showing the overlap between the SNPs identified using our signal detection approach and those identified as important by a Random Forests classifier.
