# Correction: Ramzan F. et al. “Combining Random Forests and a Signal Detection Method Leads to the Robust Detection of Genotype-Phenotype Associations” Genes, 2020, *11*, 892

**DOI:** 10.3390/genes11101199

**Published:** 2020-10-14

**Authors:** Faisal Ramzan, Mehmet Gültas, Hendrik Bertram, David Cavero, Armin Otto Schmitt

**Affiliations:** 1 Breeding Informatics Group, Department of Animal Sciences, Georg-August University, Margarethe vonWrangell-Weg 7, 37075 Göttingen, Germany; faisal.ramzan@stud.uni-goettingen.de (F.R.); gueltas@informatik.uni-goettingen.de (M.G.); hendrik.bertram@stud.uni-goettingen.de (H.B.); 2 Department of Animal Breeding and Genetics, University of Agriculture Faisalabad, Faisalabad 38000, Pakistan; 3 Center for Integrated Breeding Research (CiBreed), Albrecht-Thaer-Weg 3, Georg-August University, 37075 Göttingen, Germany; 4 H&N International, 27472 Cuxhaven, Germany; cavero@ltz.de

The authors would like to make a correction to the published paper [1]:

1. In Section 2.2, paragraph 5, the authors would like to add a new citation [54] after [53], and a sentence at the end of this paragraph, “A similar technique was used in [54] to define window boundaries for the general analysis of genomic data.”.

2. In Figure 1, the authors would like to add a sentence at the end of the figure caption, “An analogous suite of displays is shown in [54] to present the partitioning of genomic data into windows.”.

The authors would like to apologize for any inconvenience caused. The changes do not affect the scientific results. The manuscript will be updated, and the original will remain online on the article webpage, with a reference to this correction.
