# Supplementary material for: Combining Random Forests and a Signal Detection Method Leads to the Robust Detection of Genotype-Phenotype Associations
Source: Genes (Basel). 2020 Aug 5;11(8):892. doi: 10.3390/genes11080892 (PMC7465705; doi:10.3390/genes11080892)
Supplement: Supplementary file 1 [file genes-11-00892-s001.zip › Suppl_VennDiagrams.pdf]

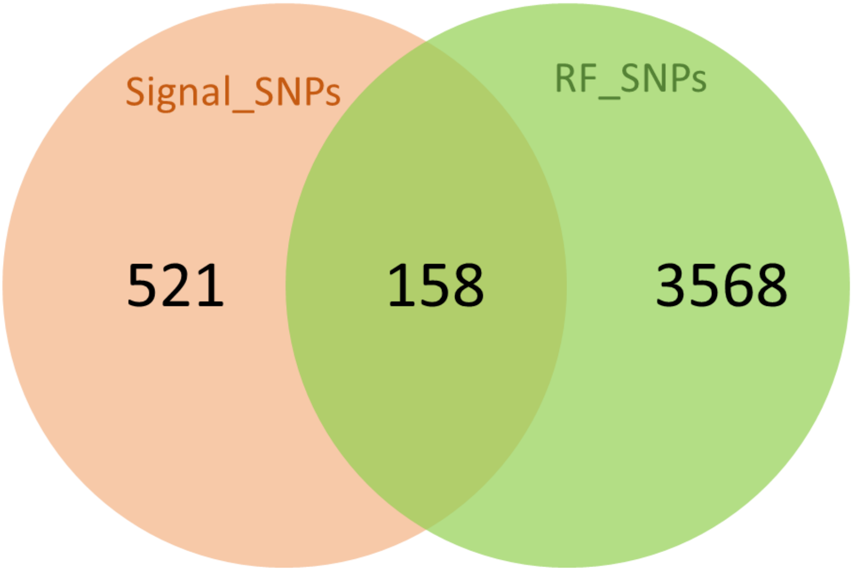

Supplementary Figure 1. Venn diagram showing the overlap between the SNPs identified for eggshell strength at time point 1 using our Signal Detection Approach (Signal_SNPs) and those identified as important by employing a Random Forests classifier (RF_SNPS).

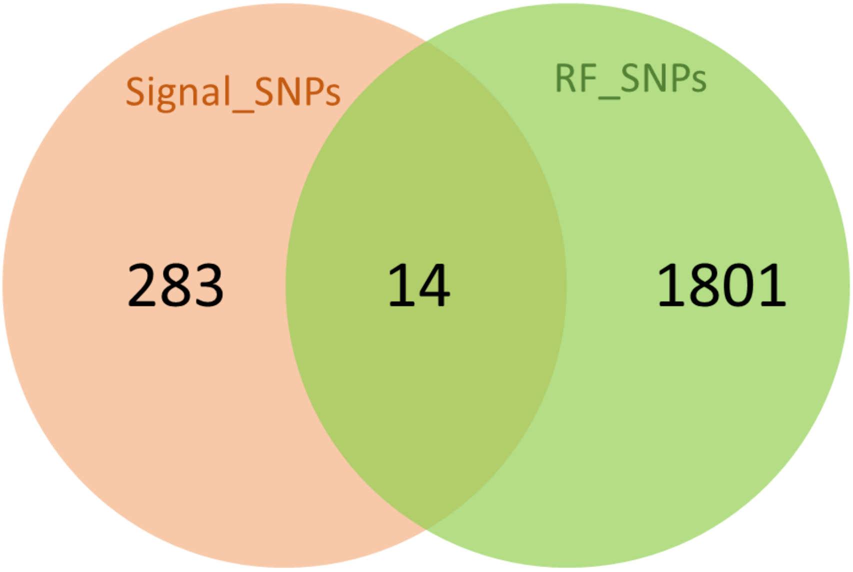

Supplementary Figure 2. Venn diagram showing the overlap between the SNPs identified for eggshell strength at time point 2 using our Signal Detection Approach (Signal_SNPs) and those identified as important by employing a Random Forests classifier (RF_SNPS).

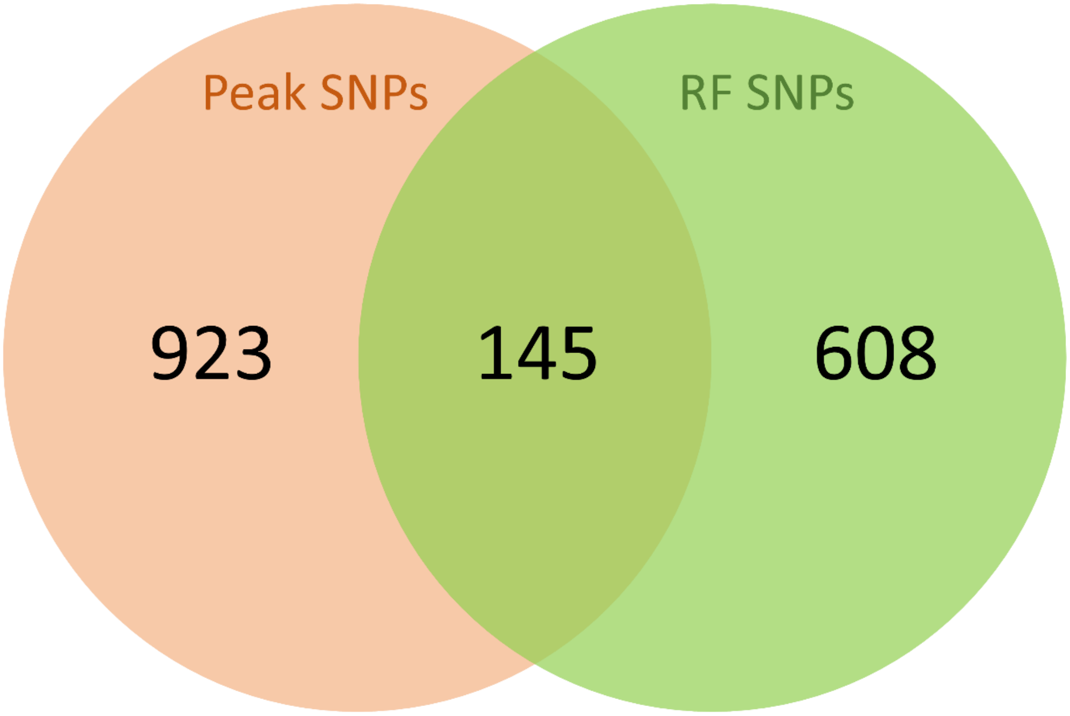

Supplementary Figure 3. Venn diagram showing the overlap between the SNPs identified for egg weight using our Signal Detection Approach (Signal_SNPs) and those identified as important by employing a Random Forests classifier (RF_SNPS).
